# Like parent, like child? Dietary resemblance in families

**DOI:** 10.1186/s12966-018-0693-1

**Published:** 2018-07-03

**Authors:** Henna Vepsäläinen, Jaakko Nevalainen, Mikael Fogelholm, Liisa Korkalo, Eva Roos, Carola Ray, Maijaliisa Erkkola, on behalf of the DAGIS consortium group

**Affiliations:** 1 Department of Food and Nutrition, University of Helsinki, P.O. Box 66, FI-00014 Helsinki, Finland; 2 Health Sciences/Faculty of Social Sciences, University of Tampere, Medisiinarinkatu 3, FI-33014 Tampere, Finland; 3 Folkhälsan Research Center, Topeliuksenkatu 20, 00250 Helsinki, Finland; 4 Department of Public Health, University of Helsinki, P.O. Box 20, FI-00014 Helsinki, Finland

**Keywords:** Dietary similarity, Dietary concordance, Familial aggregation, Whole diet, Families, Role modeling, Food environment, Social food environment, Multivariate similarity measure

## Abstract

**Background:**

Studies investigating dietary resemblance between parents and their children have gained mixed results, and the resemblance seems to vary across nutrients, foods, dietary-assessment tools used, and parent-child pairs. We investigated parent-child dietary resemblance using a novel approach in applying statistical analysis, which allowed the comparison of ‘whole-diet’ between parents and their children. Additionally, we sought to establish whether sociodemographic factors or family meals were associated with dietary resemblance and whether parent-child dietary resemblance was dependent on the parent providing food consumption data on behalf of the child (father or mother, “the respondent”).

**Methods:**

The DAGIS study investigated health behaviors among Finnish preschoolers using a cross-sectional design. One parent filled in a food frequency questionnaire (FFQ) measuring the child’s food consumption outside preschool hours during the last week. In addition, we instructed both parents or legal guardians, should the child have two, to fill in a similar FFQ regarding their own food use. Parents also reported their educational level, the number of children living in the same household, and the number of family meals. As a measure of dietary resemblance between a parent and a child, we computed Spearman correlations ranging mostly from no resemblance (0) to complete resemblance (+ 1) between parent-child pairs over the ‘whole-diet’ (excluding preschool hours). These resemblance measures were further investigated using linear mixed models.

**Results:**

We obtained 665 father-child and 798 mother-child resemblance measures. Mother-child resemblance was on average 0.57 and stronger than father-child resemblance (0.50, *p* < 0.0001), which was explained by a parent-respondent interaction: the diet of the child resembled more the diet of the parent who provided food consumption data for the child. In univariate models, father- and mother-reported number of family meals were positively associated with father-child and mother-child resemblances. Mother-reported number of family meals was positively associated with mother-child resemblance in a full model.

**Conclusions:**

The diet of the child seems to resemble more the diet of the parent responsible for the reporting of food consumption. Studies should report who provided the food consumption data for the child and take this into account in analyses, since reporter-bias can influence the results.

**Electronic supplementary material:**

The online version of this article (10.1186/s12966-018-0693-1) contains supplementary material, which is available to authorized users.

## Background

Since dietary habits, such as vegetable and fruit intake or food behavior in general, are usually learned during childhood and may track into adulthood [1–3], childhood is a crucial time to influence these behaviors. Individual factors, such as age, gender and genetic tendency to food neophobia, are not the only factors affecting food behavior: the physical and social environments are also important [4–6]. For a child, the home environment is of special significance: parents can control the availability and accessibility of foods (physical environment) as well as create social norms, offer social support and act as role models regarding healthy eating during family meals (social environment).

The social home food environment as a determinant for healthy eating among children has been widely studied, and one of the most used measures for social home food environment is parental food behavior. For example, parents’ food intake has been found to consistently be related to that of their children [7, 8]. However, studies investigating parent-child dietary resemblance have traditionally focused on nutrient intakes (e.g., fat, cholesterol, or energy intake) [9–14] or selected food or food group consumption (e.g., fruit and vegetables, high-fat foods, or beverages) [12, 15–21]. To our knowledge, only a few studies have examined parent-child resemblance in a broader sense: Vollmer et al. found a positive relationship between fathers’ and their 3–5-year-old children’s overall dietary quality as measured with the Healthy Eating Index (HEI) [22], whereas Beydoun & Wang reported only a weak correlation in HEI_n_ between parents and their 2–18-year-old children [12]. Studies investigating parent-child dietary resemblance in data-driven dietary patterns or whole-diet are also scarce. Some familial dependence on dietary clusters was observed in a sample of Finnish children aged 6 years and their mothers [23]. A recent paper reported important similarities in food consumption among families: 6–16-year-old children shared almost identical dietary patterns with their mothers, and significant similarity was also detected between the children and their fathers [24]. However, the study included the participation of 1662 mother-child dyads and 789 father-child dyads, but only 362 families provided information on both the mother and the father [24].

According to a meta-analysis published in 2011, parent-child dietary resemblance seems to vary by assessment methods used, nutrients assessed, and parent-child pairs (mother-child or father-child) in question [25]. Indeed, some studies have suggested that dietary resemblance is stronger between mothers and children than between fathers and children [10, 12, 14, 26], although not all studies have confirmed this finding [27]. Wang et al. also suggested that the age of the participating children may be associated with parent-child dietary resemblance [25]. In addition, since family meals have been linked to healthier food behavior and/or lower BMI among children and adolescents in multiple reviews [28–30] and the frequency of family meals can serve as a proxy for family functioning [30], having family meals may be associated with parent-child dietary resemblance.

To date, the determinants of parent-child dietary resemblance are poorly understood, and, as stated by Wang et al., more studies assessing those determinants are needed [25]. Thus, we investigated parent-child dietary resemblance through a ‘whole-diet’ perspective. In this paper, we use the term ‘whole-diet’ to describe the consumption of all the foods included in the food frequency questionnaire (FFQ). However, it should be noted that only food consumption outside preschool hours was measured in the children’s FFQ. Since preschoolers are unable to report their own food intake, we also sought to study the significance of the possible reporter bias due to the reporting of food consumption by proxy. Our additional objective was to investigate sociodemographic factors and family meals possibly associated with these resemblances.

## Methods

### Study design, setting, and participants

**Fig. 1:**
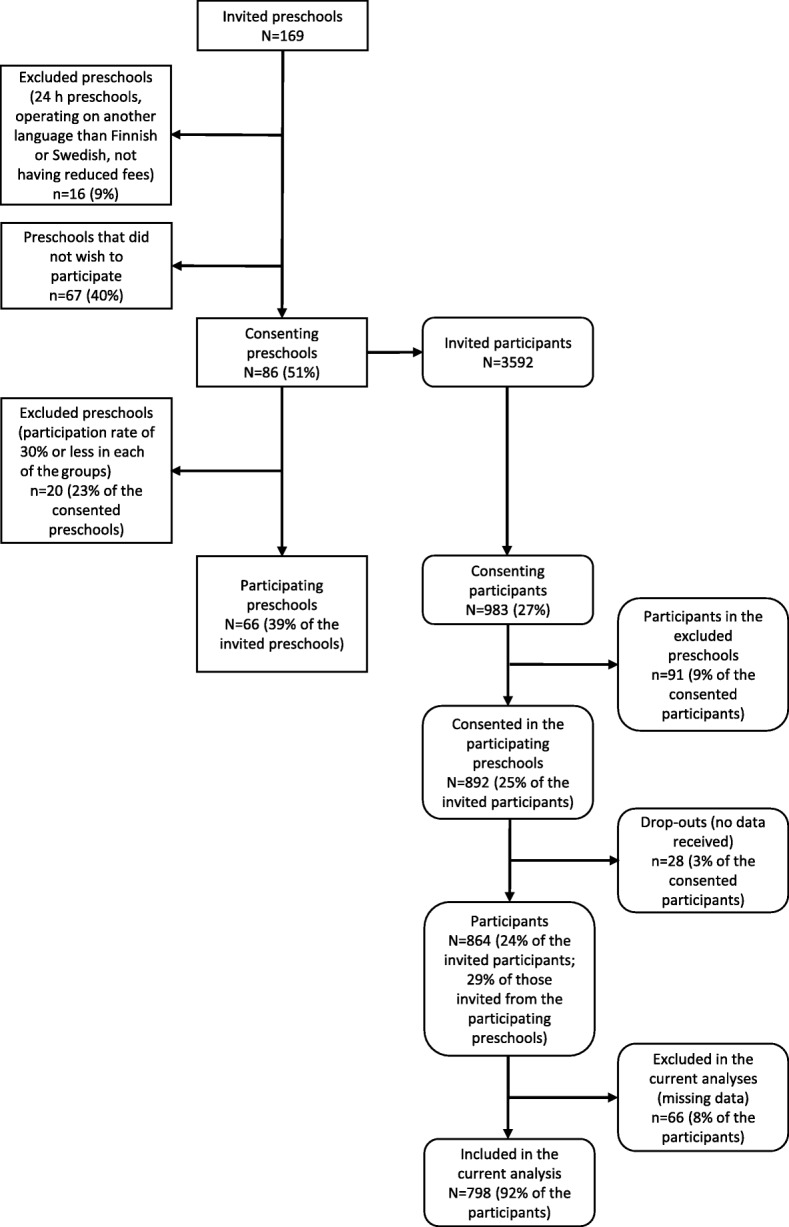
Flow of the preschools and the participants in the study

The DAGIS study (Increased Health and Wellbeing in Preschools) investigated energy balance-related behaviors among Finnish preschool children using a cross-sectional design. The DAGIS study protocol has been published previously [31]. Eight municipalities, all of which agreed, were asked to participate in the study in 2015. In order to ensure a wide range of socioeconomic backgrounds in our sample, the selection of the municipalities was based on socioeconomic status indicators (larger variation of educational level, income level and higher Gini coefficient) [32]. From all municipal preschools and private preschools from whom the municipalities purchased education services in the participating municipalities, we randomly selected 169 preschools to be invited to take part in the study. The random selection of preschools was conducted separately for each of the municipalities, and the number of invited preschools was based on power and sample size calculations [31]. Of the invited preschools, 67 (40%) did not wish to participate and 16 (9%) were excluded due to being a 24- h preschool, operating in a language other than Finnish or Swedish, or not having reduced fees for low-income families. Altogether 86 preschools (51% of those invited) provided a written informed consent. From these preschools, all the children from groups with 3–6 year-olds (*N* = 3592) and their families were invited to participate by an informational letter with a consent sheet attached. The letters were distributed through the preschools and collected from the preschools approximately one week later. The recruitment period took place between August 2015 and February 2016. Children in preschools with a low consent rate (≤30% of the children in each of the preschool groups for 3–6-year-olds consented to participate) were excluded (91 children in 20 preschools) resulting in 892 consented children (25% of those invited) and 864 participating children from 66 preschools (24% of those invited in total; 29% of those invited from the participating 66 preschools). Data collection took place between September 2015 and April 2016. Figure 1 shows the flow of the participating preschools and the participants.

### Measurements and variables

#### Dietary assessment

We used a 47-item food frequency questionnaire (FFQ) developed by the research team for the DAGIS study to measure food consumption – especially the consumption of vegetables, fruits and berries as well as sugar-enriched foods – among the participating children. The FFQ items were based on our earlier studies about the key contributors to the consumption of fruits and vegetables as well as the intake of added sucrose among Finnish children [33, 34]. Seven food groups were included in the FFQ: vegetables, fruit and berries – ten items; dairy products – ten items; fish – one item; meat and eggs – five items; cereal products – ten items; drinks – four items and others, i.e. sweets and snacks – seven items. The parents or legal guardians of the participating children reported how many times during the past week the child had consumed different foods at home or in places other than at preschool. We also recorded the person filling in the FFQ on behalf of the child (later referred to as “the respondent”). The FFQ included three answer columns: ‘not at all’, ‘times per week’ and ‘times per day’. The instruction was to either tick the ‘not at all’ -box or to write a number in one of the other columns. We intentionally restricted the FFQ to not cover municipality-provided foods and drinks consumed during preschool hours. A similar FFQ was used to measure food consumption among the parents. In the parents’ FFQ, two additional items (sweet alcoholic drinks and other alcoholic drinks) were measured but these items were, however, excluded from the analyses. The parents’ FFQ covered all the foods eaten during the last week. We instructed the parental FFQ to be filled in by both parents or legal guardians, should the child have two. The FFQs were sent to the families by post, and after completing the FFQs, the parents returned them to the preschools, from which the research staff collected them. For the analyses, food consumption data was converted into times per week.

#### Sociodemographic factors and family meals

A parent or legal guardian of the participants reported age and gender of the participating child, the number of children living in the same household as well as both parents’ age and educational level using a questionnaire distributed through the preschools. The age of the child was recoded into two categories: lower than median and median or higher. The number of children living in the same household was recoded into three categories: the participating child being the only child in the household; one child in addition to the participating child; and two or more children in addition to the participating child. Originally, seven categories were used to report the educational level of the parents. These categories were then recoded into three levels: secondary school or lower; polytechnic degree; and master’s degree or higher. In addition, both of the parents or legal guardians reported how often their family usually gets together for at least one meal on weekdays (rarely or never; on 1–2 days of the week; on 3–4 days of the week; every weekday) and weekend days (rarely or never; on one weekend day; on both weekend days) separately. The reported family meal frequencies were converted into weekly frequencies as follows: rarely or never into 0; on 1–2 days of the week into 1.5; on 3–4 days of the week into 3.5; every weekday into 5; on one weekend day into 1; and on both weekend days into 2. The reported frequencies on weekdays and weekend days were then added together to provide a single variable describing the frequency of family meals per week.

### Statistical methods

#### Dietary resemblance

To measure the parent-child dietary resemblance, we employed a multivariate similarity measure [35] for overall food consumption. The idea of the measure was to stratify the data for every unique parent-child pair, and then reverse the role of variables and observations. Instead of the conventional way, Spearman correlation was computed across all 47 FFQ items for every bivariate observation within a parent-child pair. The resulting measure is specific for each parent-child pair and can be interpreted nicely on a continuous scale from no resemblance (0) to complete resemblance (+ 1), simultaneously incorporating data on all variables measuring food consumption. In other words, the food items were ranked by their consumption frequencies within the parent’s and the child’s FFQs separately. These rankings were then compared, and identical ranking in both FFQs yielded a resemblance measure of + 1. The consumption frequencies did not have to be identical for a parent-child pair. In case of ‘opposite’ food consumption, even inverse resemblance could in principle be observed. We also calculated a resemblance measure for each mother-father pair. The chosen resemblance measure has been used for example in measuring genetic similarities of individuals [36]. Differences in rankings between the parents and their children were also calculated for each of the foods included in the FFQ (Additional file 1: Table S1).

#### Associations between sociodemographic factors, family meals and dietary resemblance

At the next stage of the analyses, we formally investigated factors that are potentially associated with the resemblance. Each family contributes several observations (resemblance measures) to this analysis, which are likely to be dependent within the family. We accounted for this dependency by introducing family-specific random intercept terms to a linear mixed model, where the outcome variable was the resemblance measure and families clustering factors. The strategy then proceeded to modelling, in which we studied whether child’s gender or age, paternal or maternal education, number of shared meals, and respondent were associated with parent-child dietary resemblance. Father-child and mother-child dietary resemblances were explained using univariate linear mixed models (SAS version 9.4; PROC MIXED), where each of the above-mentioned variables were entered into the models separately. In the full models, all variables were entered simultaneously. We excluded parents that were not mothers, not fathers, not step- or foster parents (*n* = 3), and children whose parents had filled in the child’s FFQ together (*n* = 6). All the participants with sufficient data available were included in each of the analyses (complete case analysis). We used Student’s t- and Chi-Squared -tests to compare basic characteristics of the families with and without sufficient data for the calculation of the dietary resemblance measure. Spearman correlation coefficient cut-offs [37] were used to indicate the strength of the resemblance measure.

## Results

### Participants

Parent-child dietary resemblance was calculated for 665 father-child-pairs and 798 mother-child-pairs. For the majority of the children (*n* = 659), we were able to calculate both father-child and mother-child resemblance measures. The mothers were slightly younger and more educated compared to the fathers. The participating children had a mean age of 4.74 years (median 4.75 years, SD 0.88) and 49% of them were girls. Most of the participating children lived in a household with one other child. The vast majority of the parents providing food consumption data on behalf of the child were mothers (*n* = 733, 92% of the children included in the current analyses). Descriptive statistics of the participating parents and children are shown in Table 1.

**Table 1:** Descriptive statistics of the samples used to assess mother-child and father-child dietary resemblance

	Sample used to assess father-child resemblance	Sample used to assess mother-child resemblance
Age of the parent, years, mean (SD)	38.14 (5.46)	35.67 (4.69)
Missing, *n* (%)	42 (6)	42 (5)
Education of the parent, *n* (%)		
High	159 (25)	238 (30)
Middle	224 (35)	331 (42)
Low	260 (40)	221 (28)
Missing, *n* (%)	22 (3)	8 (1)
Family meal, days/week, mean (SD)	5.70 (1.67)	5.93 (1.57)
Missing, *n* (%)	7 (1)	5 (1)
Child’s gender, *n* (%)		
Girl	328 (49)	390 (49)
Boy	337 (51)	408 (51)
Missing, *n* (%)	0 (0)	0 (0)
Child’s age, years, mean (SD)	4.74 (0.88)	4.74 (0.88)
Missing, *n* (%)	0 (0)	0 (0)
Number of children living in the same household (in addition to the participating child), *n* (%)		
0	75 (12)	95 (13)
1	347 (56)	415 (56)
2 or more	195 (32)	231 (31)
Missing, *n* (%)	48 (7)	57 (7)
Respondent, *n* (%)		
Father	62 (9)	59 (8)
Mother	596 (91)	729 (93)
Missing, *n* (%)	7 (1)	10 (1)

*N* = 741–798 for mother-child and 617–665 for father-child pairs

The participating children for whom we could not calculate the dietary resemblance measure because of missing food consumption data, had younger mothers (Student’s t-test *p* = 0.029), as well as less educated parents (Chi-Squared test for both mother’s and father’s education *p* < 0.001). In addition, there were more boys among the excluded children compared to the participants for whom the resemblance measure was calculated (Chi-Squared test *p* = 0.016). In terms of age of the child or father, number of family meals shared, children living in the same household or respondent, the excluded children did not differ from the children included in the analyses.

### Dietary resemblance in families

On average, father-child resemblance was 0.50, whereas mother-child resemblance was 0.57 (Table 2). Father-daughter and father-son resemblances did not differ from each other. Similarly, no statistically significant differences were detected between mother-daughter and mother-son resemblances. Father-mother resemblance was calculated for 666 mother-father pairs and was on average 0.52 (95% confidence interval 0.50, 0.53). Additional file 1: Table S1 shows differences in rankings between the children and their parents for foods included in the FFQ. In general, the rankings were quite similar between the children and their parents in fruit and berry soups; sweets; fruit and chocolate porridge; skimmed milk and sour milk; as well as in crisps and popcorn. Correspondingly, the rankings appeared to differ the most in sugar-sweetened juice drinks as well as in flavored and sweetened milk- and plant-based drinks.

**Table 2:** Dietary resemblance between parent-child pairs

	Father-child pairs		Mother-child pairs		Significance for difference
	Dietary resemblance (95% confidence interval)	N	Dietary resemblance (95% confidence interval)	N	
Child	0.50 (0.48, 0.52)	665	0.57 (0.55, 0.58)	798	< 0.0001
Daughter	0.50 (0.48, 0.53)	328	0.57 (0.55, 0.59)	390	< 0.0001
Son	0.49 (0.47, 0.52)	337	0.56 (0.54, 0.58)	408	< 0.0001

**Fig. 2:**
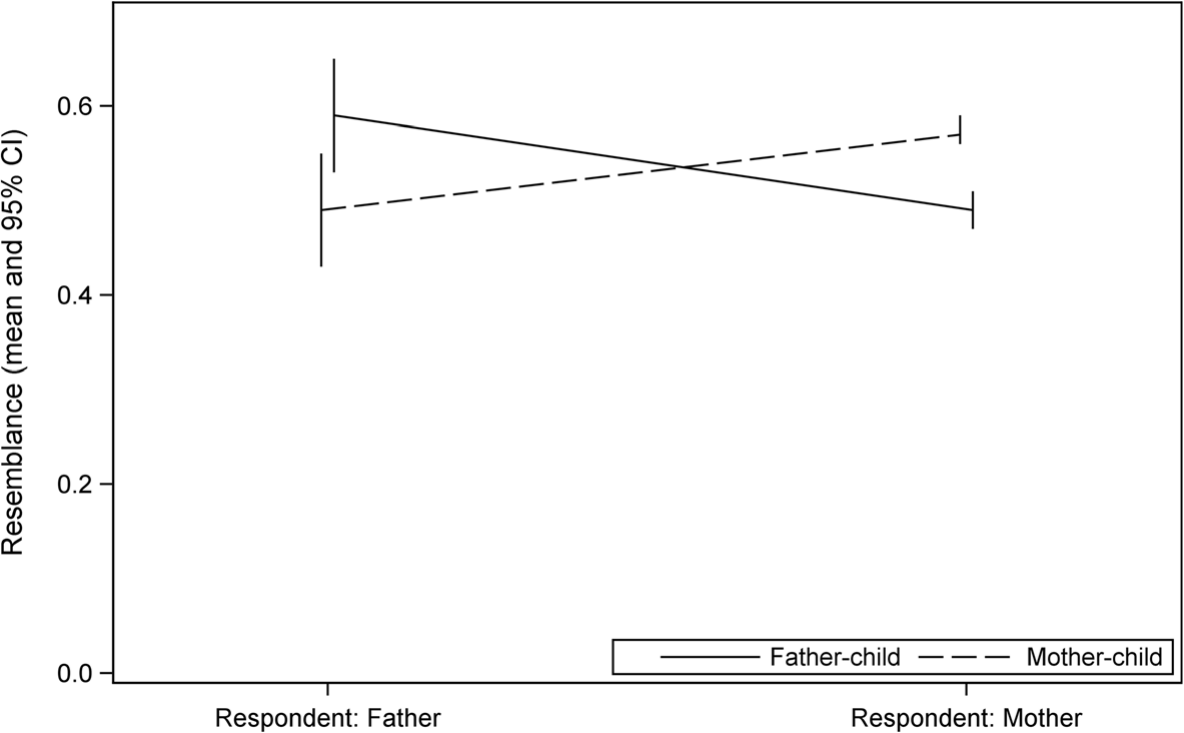
Illustration of the reporter-bias among father-child and mother-child pairs

When we set the dietary resemblance as the outcome variable with one or two observations per child, we found a statistically significant interaction between respondent (the parent providing food consumption data on behalf of the child) and the parent to whom the food consumption of the child is being compared to (*p* < 0.0001). Figure 2 illustrates this reporter-bias. In univariate models (Table 3), having mother as a respondent was negatively linked with the father-child resemblance, i.e., father-child resemblance was stronger, when food consumption data for the child was reported by the father, and weaker if the mother reported food consumption on behalf of the child. A similar reporter-bias was also detected in mother-child resemblance. In the full models (Table 4), the father-child resemblance was statistically significantly explained only by the respondent: having mother as a respondent was associated with weaker father-child resemblance. The tendency for reporter-bias was also seen in the mother-child resemblance, which was borderline significantly explained by the respondent.

**Table 3:** Unadjusted models explaining father-child and mother-child dietary resemblance, each variable entered separately into the model

	Father-child resemblance			Mother-child resemblance		
	β estimate	95% confidence interval	Significance for difference	β estimate	95% confidence interval	Significance for difference
Child’s gender						
Girl	ref.			ref.		
Boy	−0.003	− 0.029, 0.022	0.813	0.001	− 0.023, 0.025	0.927
Child’s age						
Median or higher	ref.			ref.		
Lower than median	0.005	− 0.016, 0.026	0.638	0.011	−0.009, 0.030	0.276
Number of children living in the same household (in addition to the participating child)						
Only child	ref.			ref.		
One child	0.002	−0.036, 0.040		−0.026	− 0.058, 0.007	
Two or more children	0.003	−0.036, 0.043	0.986	−0.016	−0.051, 0.018	0.277
Father’s education						
High	ref.			ref.		
Middle	−0.039	−0.085, 0.008		−0.030	−0.075, 0.016	
Low	−0.045	−0.092, 0.001	0.136	−0.039	− 0.083, 0.005	0.220
Mother’s education						
High	ref.			ref.		
Middle	−0.019	−0.062, 0.024		−0.015	−0.056, 0.026	
Low	−0.050	−0.099, 0.000	0.149	−0.034	− 0.080, 0.012	0.357
Number of family meals as reported by the father	0.014	0.003, 0.024	0.013	0.012	0.001, 0.022	0.032
Number of family meals as reported by the mother	0.018	0.007, 0.030	0.002	0.019	0.008, 0.030	< 0.001
Respondent						
Father	ref.			ref.		
Mother	−0.068	−0.129, −0.006	0.032	0.066	0.003, 0.129	0.039

*N* = 584–632 for father-child pairs and 627–717 for mother-child pairs

**Table 4:** Full models explaining father-child and mother-child resemblance, all variables entered simultaneously in the model

	Father-child resemblance			Mother-child resemblance		
	β estimate	95% confidence interval	Significance for difference	β estimate	95% confidence interval	Significance for difference
Child’s gender						
Girl	ref.			ref.		
Boy	0.003	−0.025, 0.030	0.857	0.005	−0.022, 0.032	0.719
Child’s age						
Median or higher	ref.			ref.		
Lower than median	−0.003	−0.026, 0.020	0.800	0.006	−0.016, 0.028	0.588
Number of children living in the same household (in addition to the participating child)						
Only child	ref.			ref.		
One child	0.002	−0.037, 0.040		−0.024	− 0.061, 0.013	
Two or more children	0.004	−0.037, 0.045	0.977	−0.016	−0.055, 0.023	0.418
Father’s education						
High	ref.			ref.		
Middle	−0.025	−0.078, 0.028		−0.024	−0.075, 0.027	
Low	−0.021	−0.079, 0.037	0.648	−0.036	− 0.092, 0.020	0.448
Mother’s education						
High	ref.			ref.		
Middle	−0.005	−0.055, 0.045		0.004	−0.044, 0.052	
Low	−0.030	−0.090, 0.030	0.567	−0.020	− 0.078, 0.039	0.657
Number of family meals as reported by the father	0.003	−0.014, 0.020	0.695	−0.002	−0.018, 0.014	0.819
Number of family meals as reported by the mother	0.015	−0.003, 0.034	0.109	0.022	0.004, 0.040	0.015
Respondent						
Father	ref.			ref.		
Mother	−0.090	−0.162, − 0.019	0.013	0.062	−0.007, 0.132	0.078

*N* = 574 for father-child pairs and 577 for mother-child pairs

In the univariate models (Table 3), family meals as reported by both parents separately were positively associated with father-child and mother-child resemblance. In the full models (Table 4), family meals as reported by mother was positively associated with the mother-child resemblance, whereas father-child resemblance was not explained by the number of family meals. Sociodemographic factors were not associated with parent-child dietary resemblance.

## Discussion

### Key results

Our study results demonstrate that the diets of the children and their parents were largely similar, as illustrated by the moderate resemblance. Mother-child resemblance seemed to be stronger compared to father-child resemblance, but we also found a significant parent-respondent interaction indicating that the diet of the child resembled more the diet of the parent providing responses for the child compared to the other parent. After taking the reporter-bias into account, the resemblance no longer differed between father-child and mother-child pairs. To our knowledge, this is the first study to investigate and report such an effect (i.e., stronger resemblance with the parent who reported the child’s diet), which can possibly explain some of the previous contradictory results on dietary similarities.

### Interpretation of the findings

The present study is the first dietary resemblance study to provide evidence of reporter-bias: the parent providing responses on behalf of the child might artificially strengthen the responding parent-child dietary resemblance. In former studies, mothers have traditionally provided the food intake information on behalf of their children [38]. Hence, it is unclear, whether the stronger mother-child correlations reflect the truth or if they simply reflect reporting bias and confounding, as discussed by Oliveria et al. and Shrivastava et al. [10, 14]. This view was also supported by a review of Wang et al., which detected weaker correlations in studies using child self-reported intake compared to intakes reported by parents [25]. However, mothers may be more willing to report the child’s food consumption because they are, in fact, more aware of the child’s diet. For example, in a study by Blissett et al., mothers reported greater feeding responsibility than fathers: 63% of mothers were mostly responsible for their child’s feeding, whereas the corresponding proportion among fathers was only 4% [39]. In future studies, child self-report of food consumption should be preferred whenever possible: it has been shown, that children aged 8–11 years can most accurately report their energy intake using an FFQ when compared to their parents [40]. If, however, child self-report is not possible due to the age of the participants, researchers should report clearly who provided the food consumption information and adjust for this confounding factor in the subsequent analyses. Since different family members have distinctive insights into child behavior, as discussed by Morgan et al., the two caregivers could both report the child’s diet [41].

In our study, the parent-child dietary resemblance on a ‘whole-diet’ level was relatively strong. Earlier studies have investigated dietary resemblance by calculating parent-child correlations for the consumption of different food groups. Thus, comparison between studies is challenging. Beydoun & Wang investigated similarity in dietary quality and reported a parent-child correlation of 0.26 in HEI_n_ [12]. However, HEI_n_ is designed to measure dietary quality and includes the intake of 12 food groups, whereas our approach was broader and we included a much wider range of foods, both healthy and unhealthy, covering the ‘whole-diet’ of the participants and their parents. Interestingly, a recently published paper suggested that parent-child similarity may be stronger in healthy food consumption than in unhealthy food consumption [27]. However, our data did not support this conclusion: no apparent pattern in the differences of the rankings was detected. Furthermore, our stronger resemblance can partly be related to the age of the participants: according to a review by Wang et al., younger children (< 10 years of age) have stronger correlations with their parents than older children [25]. Our results support this view: participants were preschool-aged children and thus probably more dependent on their parents’ food choices than older children are. Interestingly, studies using foods or food groups have reported slightly higher correlations than studies reporting similarities in dietary quality (HEI): in an Australian study, unadjusted father-child correlations ranged from 0.31 (fruit juice) to 0.54 (cookies) [20], whereas a study from the U.S. reported adjusted mother-child correlations ranging from 0.34 (snacks) to 0.48 (vegetables) [21]. We applied a statistical method innovatively to food frequency data in order to compare parent-child resemblance over the ‘whole-diet’ instead of comparing the use of single foods or food groups.

In this sample, we did not detect statistically significant associations between parental educational level and parent-child dietary resemblance. Regardless of the socio-economic background, families appear to share a fairly uniform diet. Although many of the studies investigating parent-child similarities in dietary intake have adjusted for parental education, to our knowledge, only one has reported analyses stratified by parental education. A study by Walsh et al. suggested that the relationships between fathers’ and children’s consumption of savory snacks and take-away foods was moderated by father’s education (university-educated vs. non-university-educated) [42]. In an adult population, the educational level of the husband (male spouse) was associated with dietary resemblance in food choices between spouses in unadjusted models, but the association disappeared after adjustment for having children [43]. In terms of other socioeconomic status markers, a stronger parent-child correlation in the consumption of artificially sweetened soft drinks in families with sufficient income (≥300 on household poverty income ratio) has been reported, although no such interaction was detected regarding other food groups [12]. Additionally, Wang et al. found a stronger mother-child correlation in energy intake among working mothers compared to unemployed mothers in a sample of African-American families [44]. Thus, our study is one of the first studies to examine the association between parental education and parent-child dietary resemblance.

The more a family eats together, the more there are opportunities for the child to assimilate health behaviors, such as healthy or unhealthy food intake. In this sample of preschool-aged children, the frequency of family meals as reported by mothers was positively associated with the parent-child dietary resemblance, whereas father-reported frequency of family meals was not. This finding could be explained by fathers’ and mothers’ different interpretations of the question (‘How often your family usually gets together for at least one meal?’). In a recent European study, an opposite result was obtained: concerning the ‘sweet and fat’ dietary pattern, the odds of being allocated to the same dietary pattern with the father was higher with more meals shared, whereas no such association was detected in the mother-child similarities [24]. Oliveria et al. reported non-significant differences in parent-child correlations between parents who ate at home more frequently and those eating at home less frequently, except for mother-child cholesterol intake [10]. In our study, parents reported having a family meal on average almost six days per week. Not many studies have reported family meal frequencies in this age group: in an Australian study, 77% of the children shared dinner (the most frequently shared family meal) with at least one parent on at least five evenings per week [45], whereas in a sample of 4-year-olds, 57% of the mothers reported that at least a part of the family ate the evening meal together on six or seven evenings per week [46].

### Strengths and limitations of the present study

In our study, only 9% of the parents reporting the child’s food consumption were fathers. These fathers were more educated compared to the fathers who did not report their child’s food consumption, and they might also have some other distinctive characteristics. We also had a fairly low participation rate: less than 30% of the children from the participating preschools took part in the study, which was notably less than anticipated. In addition, our sample was generally highly educated, which might have affected our results. Furthermore, the sample for whom the dietary resemblance measure could be calculated was more highly educated compared to the excluded participants. Thus, data was not missing completely at random, which may have biased the results and weakened the contrast and power of the corresponding tests on education. In addition, our results are from a cross-sectional study making causality hard to judge: it would be logical to reason that the child’s food consumption is influenced by the parents’ food consumption and not the other way around. However, parenting also is reacting to child characteristics, and thus, the child can also influence the food consumption of the parents [47].

The FFQ used among the children was designed to measure food intake outside preschool hours, since the parents would not have been able to estimate the consumption of foods eaten at preschool. As for the parents, they also reported foods eaten outside the home (for example at work during the days). Measuring food consumption of the children only outside preschool hours tends to make the parent-child dietary resemblance stronger than it may actually be. However, we believe that the extent of the possibly overestimated resemblance is common to all participating children, as children in preschools within the same municipality are offered same meals provided by a single catering company. Therefore, this gap in the assessment of daily food consumption probably has little effect on the ranking of the food items and the subsequent analyses. Another concern is the validity of the FFQs, which is yet unknown. However, we are currently conducting a validation study against 3-day food records. To our knowledge, no validated, culturally appropriate FFQs with an emphasis on fruit and vegetable as well as sugar-enriched food intake among preschoolers were available. Since we looked at the dietary resemblance over the whole FFQ instead of focusing on one or two food items, it is unlikely that the effect of the possible misreporting is substantial. Additionally, we used Spearman rank correlation coefficient in the calculation of dietary resemblance measures and thus, the absolute food consumption frequencies were not of importance as long as the misreporting did not interfere with the within-individual ranking of the food items. A strength of the study is that we used the same FFQ to measure food consumption both among the children and their parents, albeit the parents reported their food consumption during the whole week, whereas the children’s food consumption during preschool hours was not reported.

We did not adjust for home food availability in the present study. It has been suggested that food availability in the home modifies the associations between parental and child food intake. For example, in families where the availability of fruit and vegetables is high, parental fruit and vegetable consumption has been shown to be more strongly associated with the child’s fruit and vegetable consumption compared to the families with low fruit and vegetable availability [48, 49]. Furthermore, in a recent study by Hebestreit et al., the chance of the child sharing a ‘sweet and fat’ dietary pattern with both the mother and the father were higher where soft drinks were available during meals [24]. However, since we did not focus on certain food items, but were interested in similarities in the ‘whole-diet’, adjusting for home food availability would have been challenging and could also have led to overadjusting and misinterpretation.

## Conclusions

In this sample of Finnish preschoolers and their parents, parent-child resemblance in food consumption was relatively strong. Moreover, we also found a significant parent-respondent interaction: the diet of the child resembled more the diet of the parent providing responses for the child than the diet of the other parent. In future studies, researchers should report lucidly who provided the food consumption data for the child and take this into account in analyses, since this can influence the results.

## Additional file

Additional file 1: **Table S1.** Mean and standard deviation of differences in rankings between the children and their parents for foods included in the FFQ (negative difference: the food items was ranked higher in the child’s FFQ compared to the parent’s FFQ, and vice versa). (DOCX 21 kb)

## Abbreviations

DAGIS: Increased Health and Wellbeing in Preschools
FFQ: Food frequency questionnaire
HEI/HEI_n_: Healthy Eating Index
SD: Standard deviation
