# Supplementary material for: Like parent, like child? Dietary resemblance in families
Source: Int J Behav Nutr Phys Act. 2018 Jul 3;15:62. doi: 10.1186/s12966-018-0693-1 (PMC6031178; doi:10.1186/s12966-018-0693-1)
Supplement: Supplementary file 1 — Table S1. Mean and standard deviation of differences in rankings between the children and their parents for foods included in the FFQ (negative difference: the food items was ranked higher in the child’s FFQ compared to the parent’s FFQ, and vice versa). (DOCX 21 kb) [file 12966_2018_693_MOESM1_ESM.docx]

Table S1. Mean and standard deviation of differences in rankings between the children and their parents for foods included in the FFQ (negative difference: the food items was ranked higher in the child’s FFQ compared to the parent’s FFQ, and vice versa).

|  | **Difference in rankings between the children and their fathers** | | **Difference in rankings between the children and their mothers** | |
| --- | --- | --- | --- | --- |
| **Vegetables, fruit and berries** | **Mean** | **SD** | **Mean** | **SD** |
| Fresh vegetables | -0.15 | 5.08 | 1.21 | 4.33 |
| Cooked and canned vegetables | 1.95 | 9.65 | 3.09 | 7.93 |
| Potato | -0.47 | 5.83 | -1.72 | 5.35 |
| Peas, beans, lentils and soya | 2.99 | 8.32 | 2.60 | 6.57 |
| Fresh fruit | -3.69 | 7.05 | -1.05 | 5.29 |
| Canned and frozen fruit | 1.77 | 7.86 | 1.55 | 6.68 |
| Berries | -1.83 | 8.72 | 1.49 | 8.26 |
| Dried fruit and berries | -0.79 | 8.36 | -0.69 | 7.62 |
| Commercial baby foods and smoothies | -2.53 | 8.41 | -0.64 | 7.61 |
| Berry and fruit soups | -0.14 | 9.05 | 0.04 | 7.94 |
| **Dairy products** | **Mean** | **SD** | **Mean** | **SD** |
| Skimmed milk and sour milk | 0.32 | 12.42 | -0.14 | 11.01 |
| 1% fat milk, semi-skimmed milk and sour milk | -2.04 | 13.68 | -2.21 | 12.13 |
| Whole milk and sour milk | 0.23 | 8.20 | 0.55 | 6.37 |
| Low-fat cheese | 2.55 | 11.43 | 2.60 | 9.52 |
| High-fat cheese | 3.94 | 10.34 | 3.09 | 9.10 |
| Flavored and sweetened milk- and plant-based drinks | -4.36 | 10.57 | -4.4 | 9.47 |
| Natural yogurt and quark | 2.91 | 11.21 | 6.76 | 10.59 |
| Flavored yogurt and quark | -3.23 | 10.79 | -4.6 | 10.23 |
| Puddings | -2.45 | 8.33 | -2.65 | 7.50 |
| Ice cream | -2.00 | 7.81 | -2.34 | 7.04 |
| **Fish, meat and eggs** | **Mean** | **SD** | **Mean** | **SD** |
| Fish dishes and fish products | 1.48 | 7.28 | 1.22 | 6.35 |
| Red meat | 0.88 | 5.74 | -1.12 | 5.29 |
| White meat | 1.31 | 7.01 | 0.55 | 5.87 |
| Cold cuts | 5.51 | 10.86 | 2.22 | 10.45 |
| Sausages, frankfurters and luncheon meats | 1.37 | 9.15 | -2.14 | 7.84 |
| Egg | 3.33 | 8.02 | 2.86 | 7.34 |
| **Cereal products** | **Mean** | **SD** | **Mean** | **SD** |
| Brown rice and pasta | -1.20 | 8.31 | 0.15 | 5.93 |
| White rice and pasta | 0.26 | 8.2 | -0.65 | 6.49 |
| Rye bread, crispbread and thin rye crackers | 1.98 | 9.86 | 1.03 | 8.55 |
| Wholemeal bread | -2.03 | 9.67 | -1.83 | 8.21 |
| White bread | 1.03 | 10.23 | -0.94 | 7.76 |
| Sugar-sweetened cereals and muesli | -3.83 | 10.05 | -3.65 | 9.51 |
| Berry, fruit and chocolate porridge | -0.25 | 6.95 | 0.38 | 5.41 |
| Wholegrain porridge and cereals | -4.04 | 10.99 | -1.54 | 10.64 |
| Sweet biscuits and cereal bars | -2.44 | 8.8 | -1.57 | 7.92 |
| Sweet pastries | 0.64 | 7.93 | 0.78 | 6.53 |
| **Drinks** | **Mean** | **SD** | **Mean** | **SD** |
| Sugar-sweetened juice drinks | -5.76 | 10.36 | -6.77 | 8.82 |
| Fruit juice | 2.73 | 10.34 | 1.56 | 9.03 |
| Soft drinks | 3.34 | 8.92 | 1.75 | 7.35 |
| Reduced sugar juices and soft drinks | 3.24 | 9.42 | 2.98 | 8.17 |
| **Others** | **Mean** | **SD** | **Mean** | **SD** |
| Chocolate | 2.00 | 8.32 | 3.96 | 8.00 |
| Sweets | -0.20 | 7.73 | -0.24 | 7.29 |
| Added sugar, honey or syrup | 0.24 | 12.02 | 1.36 | 10.63 |
| Jams, marmalades and sweetened spreads | -1.68 | 9.08 | -1.26 | 8.59 |
| Plain nuts, almonds and seeds | 2.64 | 9.05 | 5.58 | 9.01 |
| Flavored nuts, almonds and seeds | 2.42 | 7.37 | 2.19 | 6.27 |
| Crisps and popcorn | 0.33 | 7.21 | 0.21 | 5.95 |
